# Tirzepatide-Induced Biphasic Anaphylactic Reaction: A Case Report

**DOI:** 10.7759/cureus.50112

**Published:** 2023-12-07

**Authors:** Zhexiang He, Agborya N Tabe, Sohaib Rana, Kristy King

**Affiliations:** 1 Internal Medicine, Conway Regional Medical Center, Conway, USA

**Keywords:** glp-1r, gip-r, type 2 diabetes mellitus, tirzepatide, biphasic anaphylactic reaction

## Abstract

Anaphylaxis is a rapid and severe reaction to a trigger that is characterized by skin, mucosal, and cardiorespiratory changes. A minority of patients exhibit a biphasic anaphylactic reaction (BAR). Tirzepatide is a dual incretin receptor analog approved for the treatment of type 2 diabetes mellitus (T2DM). Allergic reactions to tirzepatide were reported during clinical trials, but none were severe enough to be characterized as an anaphylactic reaction. We describe a case of a BAR to tirzepatide.

## Introduction

Anaphylaxis is a rapidly progressive reaction to a trigger that is characterized by mucosal and skin changes. Cardiovascular and respiratory system compromises constitute an integral part of the clinical diagnosis of anaphylaxis. Anaphylactic reactions are known to occur in 1.6%-5.1% of patients who present to the emergency department (ED), with marked variations across the world [1]. Though foods and medications are the most common triggers of anaphylaxis, no trigger is identified in a minority of patients.

The mainstay of the management of anaphylaxis includes the administration of epinephrine intramuscularly (IM) and eliminating continuous exposure to the trigger if known [2]. Despite the resolution of symptoms with epinephrine treatment, there is a proportion of patients that go on to develop a second round of symptoms and signs of anaphylaxis, a phenomenon described as a biphasic anaphylactic reaction (BAR). This phenomenon was first described in 1927, and over the years, multiple studies have been conducted to better understand the pathogenesis and factors associated with it [3].

Tirzepatide is an incretin receptor analog that was approved by the United States Food and Drug Administration (FDA) for the treatment of type 2 diabetes mellitus (T2DM) in May 2022. Its efficacy and safety have been proven in many clinical trials. Secondary benefits of clinical interest include weight loss and improvement in liver function in patients with non-alcoholic steatohepatitis (NASH) [4]. The medication is a peptide with a fatty acid side chain. The side chain favors prolonged binding with albumin, accounting for its long half-life and once-weekly dosing [5]. Its structure also favors a greater gastric inhibitory polypeptide receptor (GIP-R) binding compared to glucagon-like peptide-1 receptor (GLP-1R) binding, and this is purported to explain its greater efficacy in T2DM management and weight loss compared to other GLP-1R monoanalogs such as semaglutide [5]. Gastrointestinal side effects are the most reported [6]. We report a case of a BAR to tirzepatide, an adverse reaction not been reported so far.

## Case presentation

The patient is a 67-year-old male with a medical history of hypertension, hyperlipidemia, T2DM, and obstructive sleep apnea. He was treated two years prior to this presentation for an allergic reaction to a new soap. The patient was on semaglutide for several years. His primary care physician (PCP) switched his medications to tirzepatide for better blood sugar control.

He reported that about 20 minutes after injecting himself with 5mg of tirzepatide, he noticed a diffuse urticarial rash, swelling of his throat, shortness of breath, wheezing, and dizziness. He also had diarrhea and fecal incontinence. The emergency medical service (EMS) was called. Per the EMS report, he took the medication at 10:45 a.m. When the EMS arrived, his vital signs were as follows: blood pressure (BP) of 92/57 mmHg, pulse rate of 97 beats per minute, respiratory rate of 20 breaths per minute, and oxygen saturation of 91%. At 11:17 a.m., the patient was administered 0.5mg of epinephrine intramuscularly. En route to the ER, the patient was administered 125mg of methylprednisolone intravenously and 50mg of diphenhydramine intravenously at 11:30 a.m. By the time he got to the ER, most of his symptoms had improved. There was no cough, chest pain, dysuria, or hematuria. At the ER, his vital signs on arrival were as follows: BP of 162/82 mmHg, pulse rate of 74 beats per minute, respiratory rate of 16 breaths per minute, oxygen saturation of 97% in ambient air, and temperature of 97.6 °F. On physical examination, his conjunctiva was pink, and his sclerae were anicteric. There was no oropharyngeal or tongue swelling observed. Cardiovascular, respiratory, and gastrointestinal system exams were unremarkable. Hives were present but had improved at the time. Laboratory results are shown in Table 1.

**Table 1:** The patient's laboratory results on arrival

Lab	Result	Normal range
White cell count	5.8	3.98-10.04K/mm^3^
Hemoglobin	12.7	11.2-15.7g/dL
Hematocrit	40.2	34.1-44.9%
Platelets	342	182-396K/mm^3^
Sodium	142	135-145mmol/L
Potassium	4.7	3.5-5.1mmol/L
Chloride	104	100-110mmol/L
Bicarbonate	24	22-32mmol/L
Blood urea nitrogen	31	9-19mg/dL
Creatinine	1.4	0.6-1.3mg/dL
Aspartate aminotransferase	19	10-42U/L
Alanine aminotransferase	24	11-36U/L
Alkaline phosphatase	115	38-126U/L
Total bilirubin	0.3	0-1.2mg/dL

The patient was observed in the ER. After two hours in the ER, his symptoms recurred, this time with a marked decrease in BP (Table 2).

**Table 2:** The patient's pulse, blood pressure, and oxygen saturation trends at the ER

Time	Pulse (beats/minute)	Blood pressure (mmHg)	Oxygen saturation (%)
12:02	74	162/82	95
12:10	76	153/80	97
12:49	79	137/67	95
13:10	80	104/55	91
13:40	94	86/53	92
14:06		63/38	
14:10	88	58/36	92
14:32	79	106/58	96
15:10	75	113/54	98

He was administered a second dose of 0.5mg of IM epinephrine. Nonresponsive to this, an epinephrine drip was started. The patient was admitted to the ICU. Intravenous diphenhydramine and methylprednisolone were administered as well. Other labs done included serum IgE, C1 esterase inhibitor, and complement CH50/CH100, which came back normal. While in the ICU, he was successfully weaned off IV epinephrine. His symptoms subsided, and his vital signs were within normal limits. The patient was discharged home the following day on a short taper of prednisone. Empagliflozin was prescribed.

## Discussion

Our patient had a classic presentation of an anaphylactic reaction as defined by the European Academy of Allergy and Clinical Immunology (EAACI) since his clinical picture was characterized by skin and mucosal changes as well as respiratory and cardiovascular system collapse occurring about 20 minutes after taking tirzepatide [7]. Tirzepatide is a novel incretin analog that acts on both GIP and GLP-1 receptors to favor glucose-induced release of insulin from the pancreas. The SURPASS trials 1-5 assessed the efficacy of tirzepatide compared to placebo, semaglutide, insulin degludec as an add-on to metformin, insulin glargine, and placebo added to titrated insulin glargine, respectively [8-12]. Though hypersensitivity reactions were noted amongst participants in the experimental arm of these trials, none had an anaphylactic reaction to tirzepatide, making this case unique.

Anaphylaxis is known to be IgE-dependent, in which prior exposure to an allergen leads to an adaptive immune response characterized by the production of allergen-specific IgE that binds to the FceRI receptors on basophils and mast cells. Upon re-exposure to the same allergen, cross-linking of IgE-bound FcεRI on these cells results in cell degranulation, releasing various mediators responsible for the clinical manifestations of anaphylaxis. On the other hand, anaphylaxis can occur without prior allergen-mediated IgE production. This is often referred to as non-allergic or IgE-independent anaphylaxis [13]. It is possible that our patient elicited an IgE-independent reaction since there was no prior exposure to tirzepatide. However, these pathophysiologic mechanisms cannot be distinguished clinically.

Retrospective analysis to assess the incidence rate of anaphylaxis in patients receiving GLP-1 receptor agonists showed all medications involved in the study (lixisenatide, exenatide, liraglutide, dulaglutide, and semaglutide) have been associated with anaphylactic reactions, though lixisenatide had the least incidence rate compared to the others [14]. Our patient was initially on semaglutide and had no reaction to it. Whether or not this primed him for a potential reaction to tirzepatide could not be ascertained. This necessitates careful consideration of alternative medications within this class category. Multiple studies have shown the benefits of tirzepatide beyond its original glycemic control in patients. Such benefits include weight loss, improvement of markers of NASH, and heart failure with preserved ejection fraction [4, 15]. Based on these combined effects, patients with anaphylaxis to tirzepatide might undergo desensitization therapy. This has been successfully done for other GLP-1R monoagonists, though not in the context of an anaphylactic reaction [16].

Our patient showed features of a BAR, characterized by the recurrence of symptoms of anaphylaxis after an initial resolution. This phenomenon is reported to occur, on average, within one to eight hours of the resolution of the initial event, though cases occurring after 72 hours have been reported [17]. As a result, it is recommended that patients who are present at the ER with anaphylaxis be observed until all symptoms and signs have resolved prior to discharge [18]. Over the years, multiple pathogenic mechanisms have been proposed to explain this phenomenon, yet none has been consistently proven to cut across every situation [17]. Lee et al. studied the risk factors for biphasic anaphylaxis and found that patients with prior anaphylaxis, those with unknown inciting triggers, and those who had the first dose of epinephrine 60 minutes or more after the onset of symptoms were significantly at increased risk of a biphasic reaction [19]. Kraft et al. analyzed 435 patients with biphasic anaphylaxis and reported that more severe disease, multiorgan involvement, reaction to peanuts/tree nuts, unknown elicitors, patients with underlying chronic urticaria, those whose reaction was associated with exercise, longer duration from exposure to onset of symptoms, and treatment with antihistaminics were more likely to have biphasic reactions [20]. In addition to these, patients who needed multiple doses of epinephrine during the initial presentation were also found to be at increased risk of biphasic reactions [18]. Our patient had an allergic reaction, characterized by rash, to a detergent two years prior to taking tirzepatide, but prior allergic reactions have not been reported as a known risk factor for BARs. Nonetheless, our patient was administered antihistamines during the pre-and in-hospital phases of his care, and this constitutes the only risk factor that might have predisposed him to such a reaction.

## Conclusions

Though tirzepatide has been proven to be efficacious and relatively safe for use in the treatment of T2DM, clinicians should be aware of its potential to elicit not only an anaphylactic reaction but also a BAR. Also, it might be prudent for clinicians to educate patients on the features of anaphylaxis and think about the availability and access to emergency health services when prescribing this medication.
